# Actinomycotic osteomyelitis of a long bone in an immunocompetent adult: a case report and literature review

**DOI:** 10.1186/s12891-019-2576-2

**Published:** 2019-05-01

**Authors:** Dong Jin Ryu, Yoon Sang Jeon, Hea Yoon Kwon, Suk Jin Choi, Tae Hoon Roh, Myung Ku Kim

**Affiliations:** 1 Department of Orthopedic Surgery, College of Medicine, Inha University Hospital, 7-206, 3rd Street Sinheung-Dong, Jung-Gu, Incheon, 400-103 South Korea; 2 Department of Infectious Disease, College of Medicine, Inha University Hospital, Incheon, South Korea; 3 Department of Pathology, College of Medicine, Inha University Hospital, Incheon, South Korea

**Keywords:** *Actinomyces*, Osteomyelitis, Pathology, Culture

## Abstract

**Background:**

Actinomycosis is a rare, chronic granulomatous disease caused by Gram-positive anaerobic bacteria that colonize the oral cavity. Cervicofacial actinomycosis is the most frequent clinical presentation of actinomycosis, but hematogenous osteomyelitis at distant sites can occur in rare instance in immunocompromised or pediatric patients, only a few cases have been reported in healthy patients. Here we described a new case of distal femur osteomyelitis caused by *Actinomyces* in an adult patient who was immunocompetent and had no predisposing factors.

**Case presentation:**

A woman aged 52 years with no history of trauma presented with severe pain, swelling, and increased local heat in the proximal area of the right knee 3 weeks after she first noticed discomfort. Magnetic resonance imaging showed persistent osteomyelitis of the distal metaphysis and diaphysis of the femur with a multifocal intraosseous abscess pocket. An incision and drainage of the abscess were conducted. The tissue culture, fungus culture, acid fast bacillus (AFB) culture, AFB smear, and tuberculosis polymerase chain reaction test results were negative. A pathologic examination confirmed the presence of actinomycosis. The patient was successfully treated with intravenous penicillin G for 8 weeks followed by oral amoxicillin-clavulanate for 6 weeks with repeated surgical debridement and drainage. After a 5-year follow up, the patient had no signs of recurring infection or complications and she had full range of movement in the affected knee.

**Conclusions:**

Although rare, actinomycotic osteomyelitis can occur in healthy people. Furthermore, actinomycotic osteomyelitis is easily misdiagnosed as tuberculosis in areas with a high prevalence of tuberculosis. To detect and identify the bacteria accurately, pathologic examination should be performed as well as culture tests, because the probability for culture confirmation of actinomycosis is quite low. The initial treatment is vital to a successful outcome without ostectomy or amputation.

## Background

*Actinomyces* is an anaerobic Gram-positive filamentous organism, commonly found at high concentrations in tonsillar crypts and gingivodental crevices. More than 30 species of *Actinomyces* have been described. *Actinomyces israelii* is the most prevalent species in human infections and is found in most actinomycosis [1]. Osteomyelitis caused by *Actinomyces* is uncommon, and located primarily in the head, neck, and cervical areas [1, 2]. Actinomycotic osteomyelitis resulting from hematogenous seeding is rarely reported, and most cases occur in children or immunocompromised patients who have predisposing factors [1, 3–5]. However, a small number of cases of actinomycotic osteomyelitis in the lower legs of healthy people, especially the long bone, have been reported. It shows variable clinical presentation and leads to diagnostic confusion, can mimic malignancy, and result in delayed therapy. In particular, in areas with a high prevalence of tuberculosis, initial treatment may fail due to misdiagnosis and therefore incorrect treatment of tuberculosis [1, 6–9]. Here we described a new case of distal femur osteomyelitis caused by *Actinomyces* in an adult patient who was immunocompetent and had no predisposing factors.

## Case presentation

A woman aged 52 years with no prior trauma presented with severe pain, swelling and increased local heat in the proximal area of the right knee. The patient’s symptoms developed 3 weeks prior to her arrival at our hospital. She initially presented with pain in both knees at the local clinic and was treated with physical therapy and hyaluronic acid injections. The left knee pain resolved following treatment. However, the right knee pain persisted, and the patient reported that the pain and increased local heat had extended to the more proximal area. Furthermore, the patient developed a high fever of over 39 °C 2 weeks after first treatment. She was referred to our hospital with a suspected distal femur bony malignancy.

The patient had no past medical history of diabetes mellitus, hypertension, hepatitis, or systemic infection. The patient’s Human Immunodeficiency Virus (HIV) test, and liver and kidney function tests were normal. Notably, she had a salphingectomy 15 years prior, and a single tooth extracted approximately 4 months prior to presentation. She received prophylactic antibiotics before the tooth extraction.

**Fig. 1:**
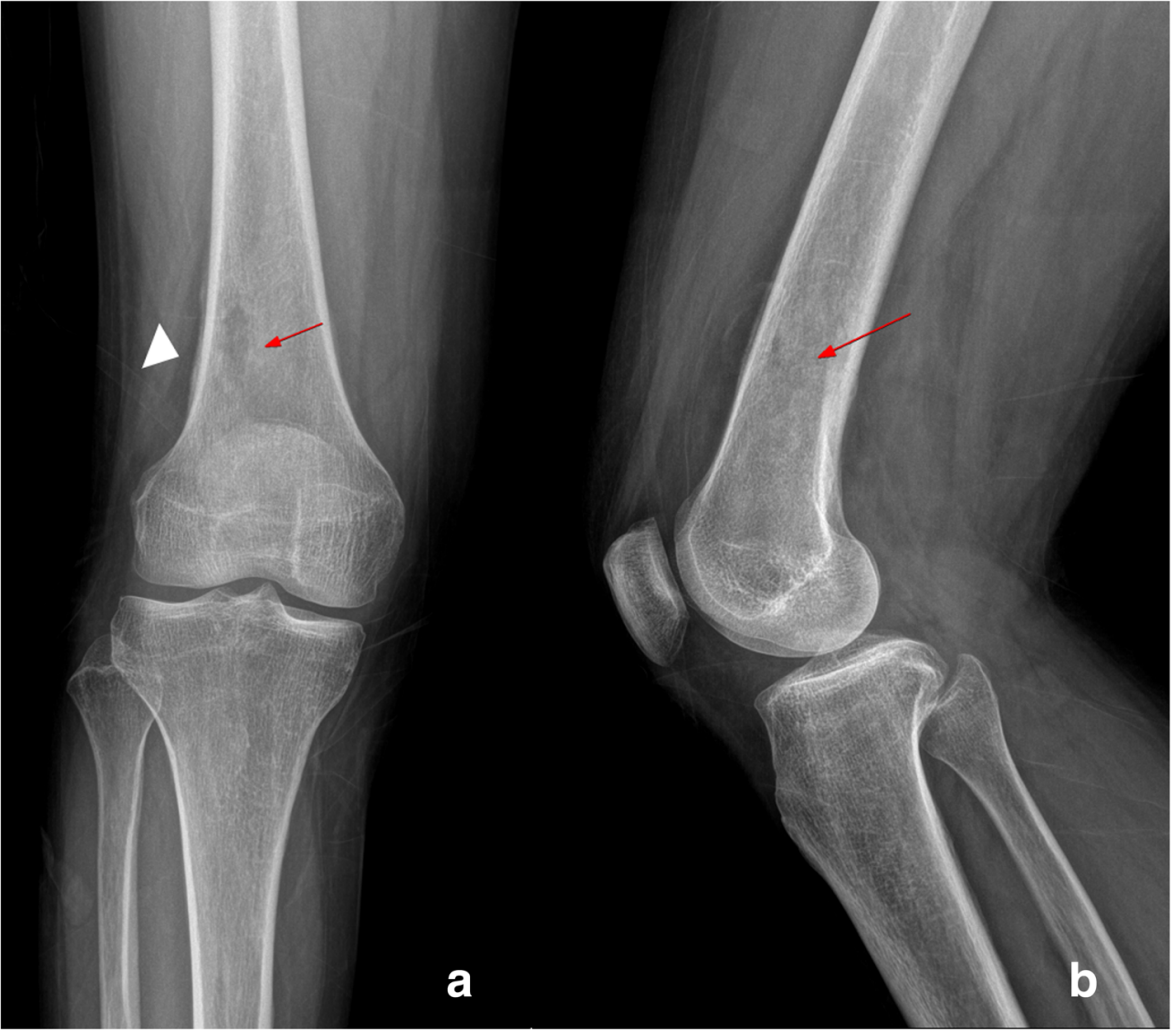
Initial X-ray findings of the right knee. Anteroposterior (**a**) and lateral (**b**) radiography of the right knee revealed multifocal osteolytic changes (arrow) in the distal metaphysis area of the right femur. There was a geographic lesion with an irregular margin, but, no sclerotic rim (Lodwick classification type 1B). There was no destruction of cortical bone. However, a subtle cortical thickening lesion suggesting solid type of periosteal reaction was found at superior aspect of lateral femoral condyle (arrow head, a)

**Fig. 2:**
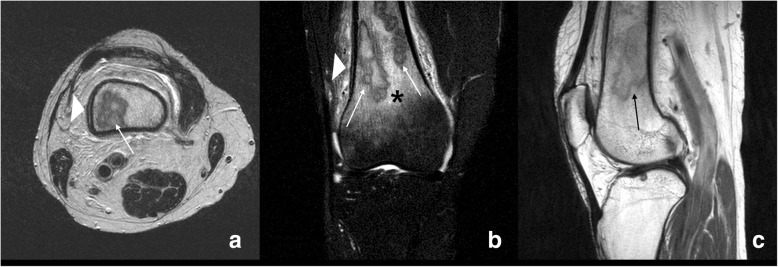
Preoperative MRI findings of the right knee. Axial (**a**), coronal (**b**), and sagittal (**c**) T2-weighted MRIs are shown. There were multifocal intraosseous lesions (arrows, 6.1 × 2 × 2 cm sized) at metaphysis and diaphysis of the distal femur. There was no destruction of cortical bone. However, double periosteal line, which suggesting periosteal reaction (arrow head, **a** and **b**), and marrow edema were found in the surrounding tissues (asterisk, **b**)

On the physical examination, an increased local heat in the proximal area of the right knee without an external wound, or draining sinus was confirmed. Body temperature was 38.8 °C. Laboratory test results showed the following: leukocytes 7260/μL (neutrophil 79.1%), absolute neutrophil count 4050, C-reactive protein (CRP) 21.26 mg/L, and erythrocyte sedimentation rate (ESR) 72 mm/h. We conducted synovial fluid analysis on the fluid extracted from the right knee joint. Synovial fluid analysis revealed a white blood cell count of 870/mm^3^, a polymorphonuclear leukocyte of 45%, and no crystals were found. Anteroposterior and lateral radiography of the right knee revealed multifocal osteolytic changes in the distal metaphysis area of the right femur. The lesion had an irregular margin but no sclerotic rim (Lodwick classification type 1B). There was no definite destruction of cortical bone. However, a subtle cortical thickening lesion suggesting solid type of periosteal reaction was found at superior aspect of lateral femoral condyle (arrow head, a) (Fig. 1). The axial (a), coronal (b), and sagittal (c) T2-weighted magnetic resonance imaging (MRI) of the right knee, which were previously performed at another hospital (with lacking information of T1 weighted and enhanced image), are shown in Fig. 2. Multifocal intraosseous lesions (6.1 × 2 × 2 cm) at metaphysis and diaphysis of the distal femur were observed with no destruction of cortical bone. However, double-contour periosteal line, which suggesting periosteal reaction (arrow head, A and B) at lateral aspect, and marrow edema were found in the adjacent tissues (asterisk, B). Considering the patient’s age, location of the lesion, margin, and absence of cortical destruction, we suspected osteomyelitis rather than a bony malignancy.

Still considering the possibility of malignancy, we made a 5 cm longitudinal incision in the anterolateral aspect of the distal femur. We split the vastus lateralis muscle and made an oval-shaped bony window in the lateral aspect of the femur. We evacuated yellowish pus-like intramedullary abscess, and the tissues were sent for Gram staining, routine culture, acid-fast bacillus (AFB) culture, fungus culture, and pathology test. Tissues were collected from various sites, and frozen pathology test was performed to exclude the possibility of malignancy. The emergency frozen pathology examination showed an absence of malignant cells. Continuous, massive marginal debridement and irrigation were performed. We inserted a negative-pressure drain into the intramedullary canal through the distal femur bony window. Intravenous first-generation cephalosporin (Cefazolin, 2 g/q8hr) was empirically used to target methicillin-susceptible *Staphylococcus aureus*, which is still the most common cause of osteomyelitis. The tissue culture, fungus culture, AFB culture, AFB smear, and tuberculosis (Tb) polymerase chain reaction (PCR) tests were negative.

**Fig. 3:**
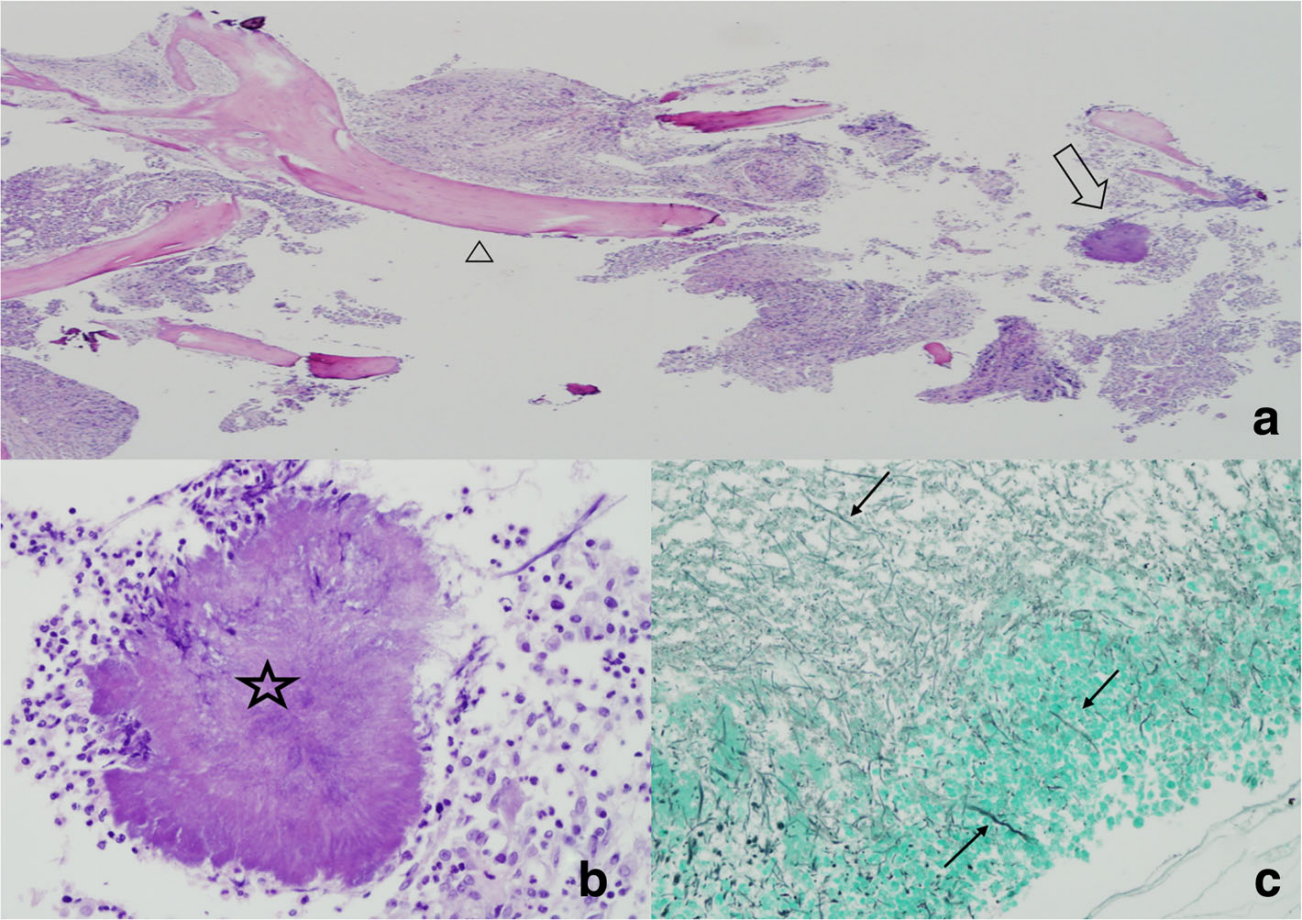
Pathologic findings of drained materials. **a** The haematoxylin-eosin stain (× 4), of the curettage specimen shows necrotic bone detritus (arrowhead) and inflamed granulation tissue consistent with osteomyelitis. We identified a sulfur granule (arrow). **b** The haematoxylin-eosin stain (× 40), shows the sulfur granule (asterisk) is rimmed by neutrophils. The granule is composed of filamentous bacteria embedded in an amorphous matrix. **c** GMS stain (× 20) shows individual bacterial filaments (arrows)

Six days post-surgery, pathologic examination of a bone biopsy specimen revealed acute and chronic osteomyelitis. Furthermore, colonies of filamentous bacteria that were rimmed by neutrophils with sulfur granules, which are indicative of actinomycotic colonies, were present. (Fig. 3) However, we were unable to confirm the exact species of *Actinomyces*. As a result, the treatment plan was altered, and intravenous penicillin G potassium (400 million IU/q6hr) was administered to target the actinomycotic osteomyelitis. The patient received repeated curettage and debridement on postoperative days 13, 27, 41, and 54 because of continuous pus- like discharge through the suction drain and abnormal ESR and CRP results. The specimens obtained on days 13, 27, and 41 were still positive for sulfur granules on pathologic examination. However, the culture tests remained negative. The specimen obtained on day 54 was normal on following pathologic examination. At postoperative day 60, the yellow pus-like discharge decreased in volume, and the ESR and CRP results were normal. The drain was removed, and the wound was closed. Penicillin G potassium was Intravenously administered for 8 weeks followed by amoxicillin-clavulanate taken orally at a dosage of 625 mg every 6 h for 6 weeks. CRP and ESR were tested at 2-week intervals. After confirming the CRP and ESR values were normal for 3 consecutive intervals and that there were no signs of recurrence (local heating, pain, swelling, or painful range of motion), we discontinued the oral antibiotics. Antibiotics were discontinued after total 14 weeks of treatment.

**Fig. 4:**
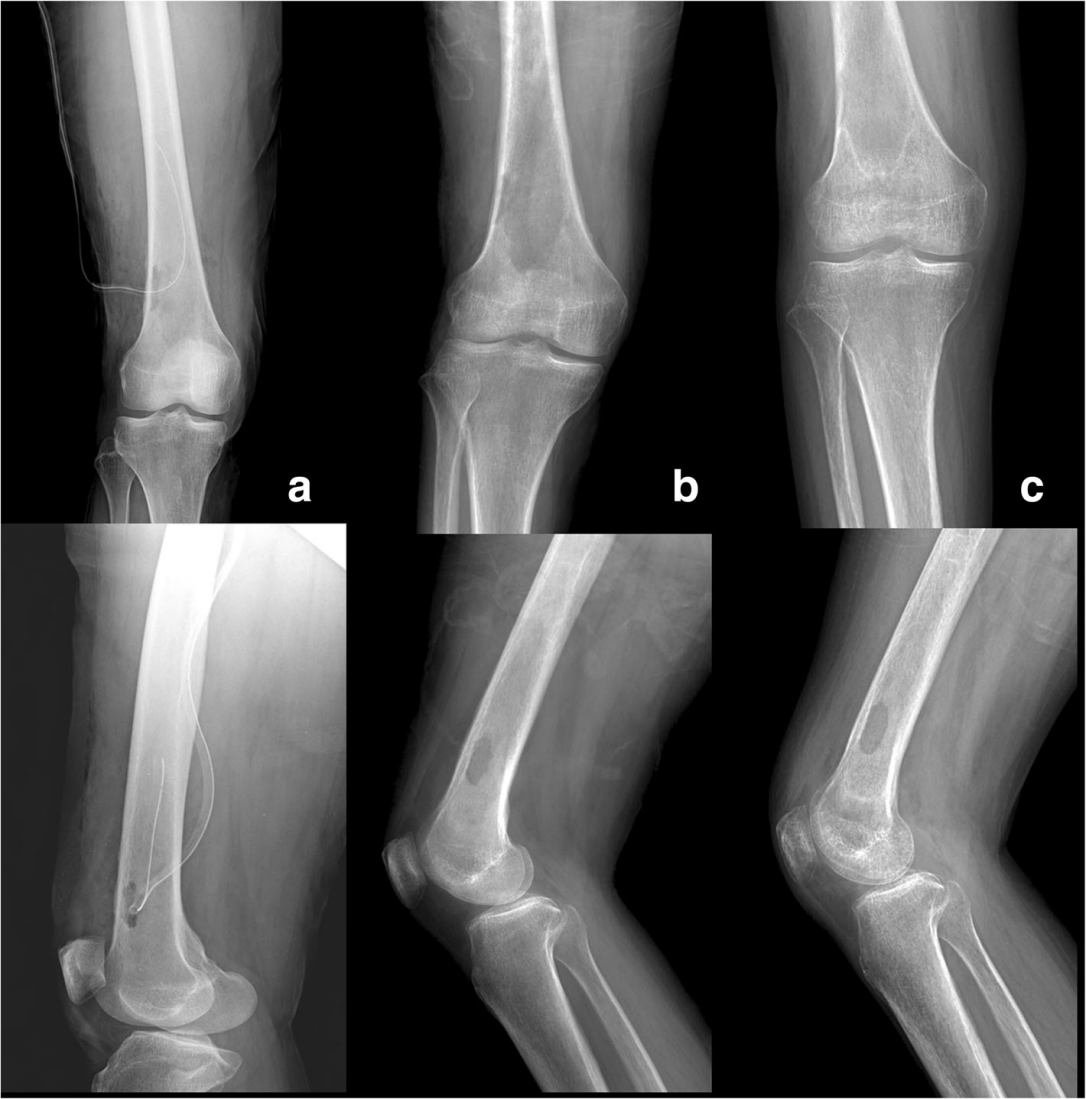
Serial X-ray findings according to progress. **a** Post-operative anteroposterior and lateral radiography of the right knee shows the oval-shaped small bony window that was made at the lateral aspect of the distal femur and reveals the drain insertion status. **b** Anteroposterior and lateral radiography after the fifth debridement shows that the bony curettage range was wider. **c** Anteroposterior and lateral radiography at the 1-year follow-up; the sclerotic rim is formed, and the bony window remains. There was no cortical destruction or periosteal reaction

Follow-up was performed at 6 months, 1 year, 3 years and 5 years after surgery. At the follow-up, X-rays showed that the bony window was not fully recovered, but there was no evidence of recurrence or pathological fracture (Fig. 4). During the 5-year follow-up period, CRP and ESR levels remained normal, and there were no symptoms of recurrence, such as local heating, swelling, and pain. In addition, the patient had full range of motion in the affected knee. The patient is satisfied with the result of treatment and enjoy daily life with no discomfort.

## Discussion and conclusions

Between the years of 1990 and 2017, 8 cases of lower leg actinomycotic osteomyelitis have been published in the English-language literature (Table 1). Osteomyelitis of the long bone is generally attribute to hematogenous spread, trauma, or the presence of a prosthetic device [10]. Anaerobic osteomyelitis is usually rare [11], and typically reported in patients with a chronic underlying disease or following a complicated bone fracture. These cases usually present without hematogenous spread. Our patient had no specific infection source or predisposing disease other than a recent single-tooth extraction. Therefore, our presumption was that the tooth extraction resulted in hematogenous seeding of *Actinomyces* to a distant site, causing the osteomyelitis. There are only a few case reports of long bone osteomyelitis following an oral infection [12].

**Table 1:** Cases of lower leg osteomyelitis caused by Actinomycosis reported in English language literature between 1990 and 2017 with our patient

Cases	Age/Gender	Location	Underlying disease	Culture result	Pathologic exam	Treatment	Outcome
Atwaru et al. [9]	32/M	Calcaneus	None	Primary: none Delayed: *Actinomyces Israelii*	Primary: none Delayed: Actinomyces	Antituberculosis medication → Penicillin (PO) 6 W	Total calcanectomy
Lee et al. [15]	59/M	Fibular	None	Actinomyces meyeri Fusobacterium nucleatum	Actinomyces	Fibular excision Ampicillin-sulbactam (IV) 3 W + Amoxicillin-clavulanate (PO) 8 W	Fibular excision
Apotheloz et al. [5]	47/M	Rt. lung Rt. Proximal tibia	Alcoholics smoking	Actinomyces meyeri	Actinomyces	Synovectomy Penicillin (IV) 8 W + penicillin (PO) 12 M	Cured
Tekin et al. [20]	90/F	Knee, ankle joint (proximal & distal tibia)	Unknown	Unknown	Actinomyces	Ampicillin/sulbactam (IV) 2 W + Amoxicillin-clavulanate (PO) 6 M	Lower leg amputation
Kundu et al. [6]	50/M	Knee joint (distal femur + proximal tibia)	Unknown	Actinomyces	Actinomyces	Repeated bone curettage Penicillin (IV) 6 W	Failed- amputation
Nayak et.al. [17]	30/M	Calcaneus	Unknown	Actinomyces MSSA	Actinomyces	Debridement/Antibead insertion ketaconazole + Tetracycline + Septran-DS (PO)6 W	Cured
Yusof et al. [7]	34/M	Knee joint, proximal tibia	Unknown	None	Actinomyces	Bactrim and amoxycillin (PO) 6 M	Cured, But ROM limitation
Nandy et al. [21]	52/M	Talus, medial malleolus	None	Unknown	Actinomyces	Clindamycin (IV) 3 W	Below knee amputation
Our patient	52/F	Distal femur	None	None	Actinomyces	Repeated bone curettage Penicillin (IV)8 W+ Amoxicillin-clavulanate (PO) 6 W	Cured

To diagnose an atypical osteomyelitis such as this case, malignancy (especially Ewing sarcoma) [13], metastatic tumor, Langerhans cell histiocytosis, and Garre’s sclerosing osteomyelitis should be considered and excluded. The age of the patient, location of the lesion, clinical features, degree of progression, soft tissue reaction, existing sinus, and radiological findings (X-ray, CT, MRI, bone scan) should be considered together. In this case, because of the more suspecting osteomyelitis in the pre-exam, we started with two plans. First, biopsy would be conducted by a mini-open lateral approach, and when malignancy cells appeared in the frozen section, end the operation and prepare for the second bony malignancy operation. But in the actual operation field, there was definite pus-like abscess drainage and no cells suspected of malignancy in multiple frozen sections. After that, we conducted massive debridement and irrigation. However, because of the possibility of diagnostic errors in the emergency test through the frozen test [14], it would be more safe to confirm the biopsy exam and establish a final treatment plan.

Actinomycotic osteomyelitis can easily be mistaken for more common conditions with discharging sinuses, such as pyogenic osteomyelitis and tuberculosis [1], especially in areas with a high prevalence of tuberculosis. As a result, initial treatments may use an anti-tuberculosis agent due to a suspected misdiagnosis of tuberculosis osteomyelitis [1, 9]. The incubation period until the symptoms are manifested after Actinomycosis infection has not been clearly established. It is known to be very diverse, and at literature review of current study show that it vary from three months [15] to as long as 15 years [9].

Culture confirmation of the infectious organism is important for the development of an effective treatment strategy. Prompt transport of the specimens to the microbiology laboratory, preferably n anaerobic medial, is necessary for optimal isolation. However, *Actinomyces* is difficult to culture. Reiner et al. [16] could only confirmed 17 cases from a total of 35 suspected cases by culture. Thus, pathologic examination is vital to an accurate diagnosis. For our patient, the routine cultures, fungus culture, AFB culture, and Gram staining were negative. However, we were able to make the correct diagnosis by confirming the presence of sulfur granules on pathology examination. If there are atypical clinical findings or unexpected operative findings, we recommend culture, gram staining, Tb PCR, and pathologic examination. Early diagnosis and proper treatment for actinomycosis is important for a successful outcome and to prevent persistent draining sinus, deformation, sclerotic change of bone, and lower-limb amputation [6, 8, 17].

*Actinomyces* is extremely susceptible to beta-lactams, especially penicillin G or amoxicillin, and drug resistance is not considered to be a problem. Patients require many times of drainage and debridement until the signs of infection are no longer present and the pathologic examination become negative. In addition, prolonged (at least 4 to 8 weeks) high doses of penicillin G or amoxicillin are needed. Notably, the antibiotic duration could likely be reduced for patients in whom optimal surgical resection of the infected tissue has been performed. Reiner et el. [16] suggested that surgical therapy was necessary in most instances. We did not use in our patient, Nayak et el. [17] reported that using bone cement (antibead) with a mixture of antibiotics can increase the local therapeutic effect. However, penicillin G is not stable at the heat which generated during the process of making antibiotic cements. Therefore, amoxicillin would be a better choice for the antibiotic cement [18]. Vancomycin is also known to be effective antibiotics in Actinomyces management [19]. In general, osteomyelitis due to Methicillin-susceptible *Staphylococcus aureus* or Methicillin-resistant *Staphylococcus aureus* is common, thus insertion of vancomycin loaded bone cement may be a good initial management until the final culture exam result.

Pending the results of culturing, a lesion with granules of branching Gram-positive bacteria should be empirically treated with penicillin and a sulfonamide. Actinomycotic osteomyelitis has a high rate of successful treatment outcomes, but several additional surgical treatments may be needed [6, 15]. Kundu et al. suggested that a delayed diagnosis leads to sclerosis of the bone, which hampers the penetration of penicillin and makes infection control more difficult [6]. In the previously reported case, partial ostectomy or amputation was performed in 3 out of 8 patients (37.5%) [6, 9, 15, 20]. Our patient was successfully treated with repeated marginal debridement and drainage without the need for ostectomy or amputation. The pus-like discharge in our patient persisted despite the IV antibiotics, open drainage, and wound care. Consequently, a total of five debridement and bone curettages were performed and the duration of antibiotic usage was quite long, with 8 weeks for intravenous antibiotics and 6 weeks for oral antibiotics.

Actinomycosis is an extremely rare and challenging disease for orthopedic surgeons and can be easily mistaken for more common conditions with discharging sinuses, such as pyogenic osteomyelitis and tuberculosis. This is especially common in areas with a high prevalence of tuberculosis. Routine culture and Gram staining are still important, but the culture confirmation rate is low. Therefore, the lessons learned from this case are that it is important to make an accurate diagnosis early on by carrying out the procedure in accordance with various exam (including pathology, Tb PCR, culture test), even if it is a typical or atypical lesion. With a prompt diagnosis and initial treatment, actinomycotic osteomyelitis can be successfully treated without ostectomy or amputation.

## Abbreviations

AFB: Acid-fast bacillus
IV: Intravenous
PCR: Polymerase chain reaction
ROM: Range of motion
Tb: Tuberculosis
